# Hemophagocytic lymphohistiocytosis and myopericarditis induced by campylobacter: a case report

**DOI:** 10.1186/s12879-024-09128-z

**Published:** 2024-04-08

**Authors:** Chia Hua Chang, Chih-Chuan Kao

**Affiliations:** 1 Department of Internal Medicine , Tungs’ Taichung MetroHarbor Hospital, Taichung City, Taiwan; 2 Department of Infection, Tungs’ Taichung MetroHarbor Hospital, No.699, Section 8, Taiwan Boulevard, Wuqi District, Taichung City, 43503 Taiwan

**Keywords:** Hemophagocytic lymphohistiocytosis, Myopericarditis, *Campylobacter*

## Abstract

**Background:**

Hemophagocytic lymphohistiocytosis (HLH) is a severe disorder characterized by excessive activation of the immune system, leading to hypercytokinemia and damage to multiple organs. We report a rare case of HLH with myopericarditis caused by *Campylobacter* infection.

**Case presentation:**

A 28-year-old male patient with a history of hypertension without medicine control presented at the hospital after a four-day fever, decreasing urine amount, rashes on his trunk and limbs, and other symptoms. He was admitted with a provisional diagnosis of atypical infection and allergic skin rash related to diclofenac. However, his condition deteriorated, and he developed shock, tachycardia, chest distress, and bilateral pleural effusion after admission. Further investigations revealed cardiogenic shock related to myopericarditis, and he was transferred to the ICU. In addition, a stool PCR panel subsequently revealed a positive result for *Campylobacter*. On day 6, he was diagnosed with HLH. Under Clarithromycin and dexamethasone infusion, leukocytosis, anemia and thrombocytopenia with cardiogenic shock status improved. Then, he was later discharged in stable condition.

**Conclusions:**

HLH and myopericarditis caused by *Campylobacter* are very rare. Early detection of *Campylobacter*-induced HLH and multiple organ failure, as well as prompt use of antibiotics and immunosuppressants, can be helpful for prognosis.

**Supplementary Information:**

The online version contains supplementary material available at 10.1186/s12879-024-09128-z.

## Background

*Campylobacter spp.* are gram-negative, short rods that thrive under microaerophilic conditions and do not form spores [1]. The most common sources of *Campylobacter* infection in humans are poultry, followed by beef and mutton [2]. *Campylobacter* is a widespread cause of bacterial gastroenteritis worldwide. Aside from gastroenteritis, *Campylobacter* may also cause autoimmune conditions, such as Guillain‒Barré syndrome, reactive arthritis, and irritable bowel syndrome [3]. Although infrequent, myopericarditis due to *Campylobacter* has been reported in some case reports [4]. Conversely, there is only one report of HLH caused by *Campylobacter* [5]. We present a rare case of myopericarditis concurrent with HLH caused by *Campylobacter*.

## Case presentation

In June 2022, a 28-year-old man was referred to our hospital due to a four-day fever accompanied by a decrease in urine output. The patient had a history of hypertension but was not currently taking any medication to control it. He worked at a textile factory and had a part-time job at a poultry slaughterhouse. Three days before presenting to our hospital, the patient visited a local clinic for symptoms such as fever, headache, sore throat, epigastric pain, and diarrhea. The doctor at the clinic prescribed medications, including diclofenac and loperamide, to relieve the symptoms. However, one day later, the patient experienced vomiting, persistent fever, and new rashes on his trunk and limbs, which were not itchy, accompanied by a decrease in urine volume. Therefore, he returned to the clinic and was subsequently referred to our hospital.

**Fig. 1:**
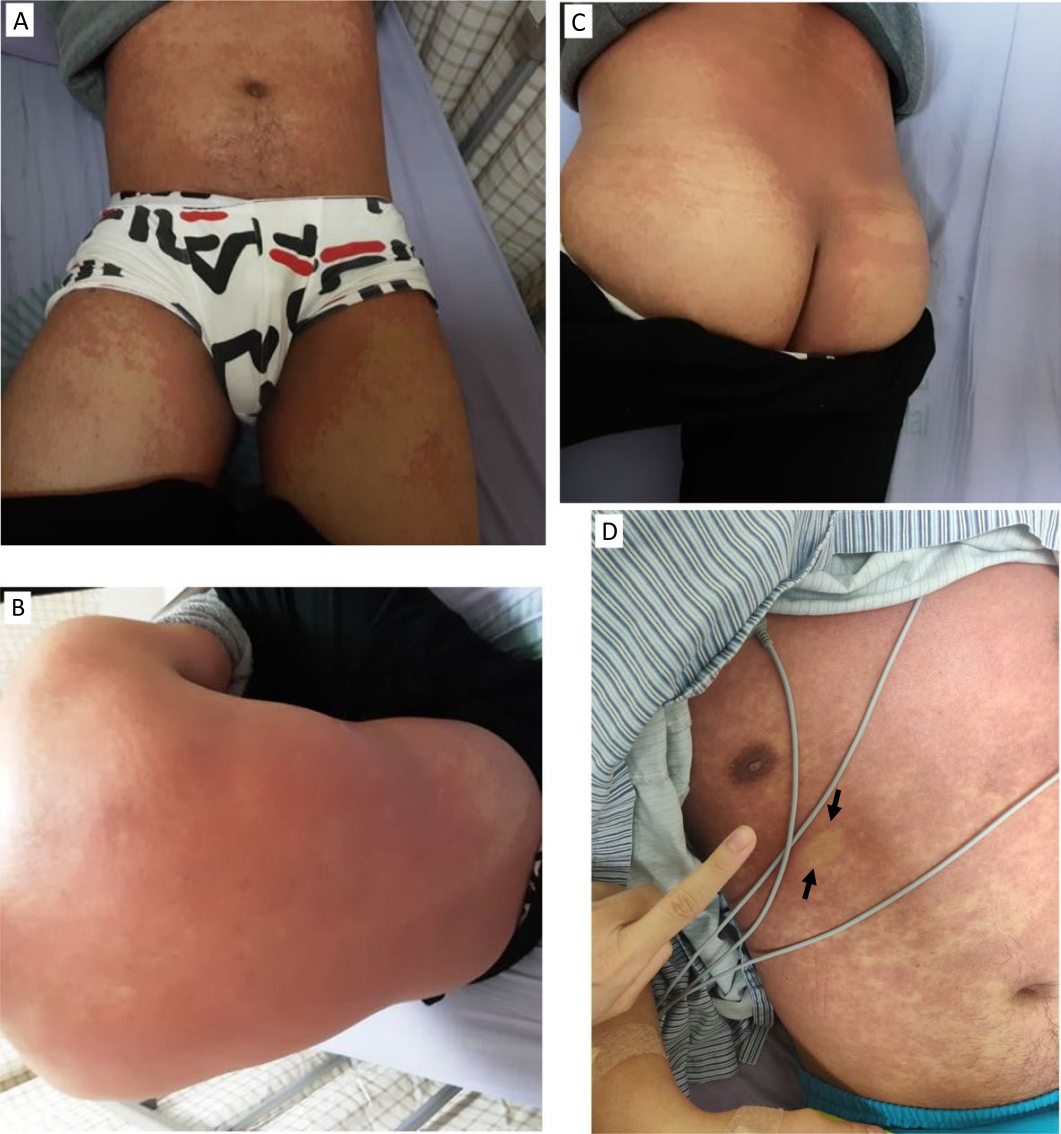
Skin rash during admission

Upon examination in our emergency department, the patient had a pulse rate of 130 beats per minute, a blood pressure of 142/63 mmHg, a temperature of 38.6 °C, and an oxygen saturation of 98% on room air. We observed icteric sclera and a fused, blanchable macular rash without itching on the trunk and all four limbs without mucous membrane involvement. (Fig. 1). The physical examination on the day of admission revealed a fused-macular rash distributed across the anterior and posterior trunk as well as the bilateral lower legs (Panels A, B, C). Additionally, a blanchable skin rash is present on the anterior chest, as indicated by the arrow (Panel D).) Laboratory studies showed a white blood cell count of 7100/µL, a creatinine level of 1.15 mg/dL, an elevated aspartate aminotransferase level of 66 IU/L, an alanine aminotransferase level of 104 IU/L, a total bilirubin level of 2.4 mg/dL, a direct bilirubin level of 1.2 mg/dL, a C-reactive protein level of 19.03 mg/dL (normal range < 0.4 mg/dL), and a procalcitonin level of 1.3 ng/mL (normal range 0–0.5 ng/mL) (Laboratory data collected during the hospital stay are recorded in Additional file 1: Table 1–3). We admitted the patient with a diagnosis of an atypical infection and a diclofenac-related allergic skin rash.

**Fig. 2:**
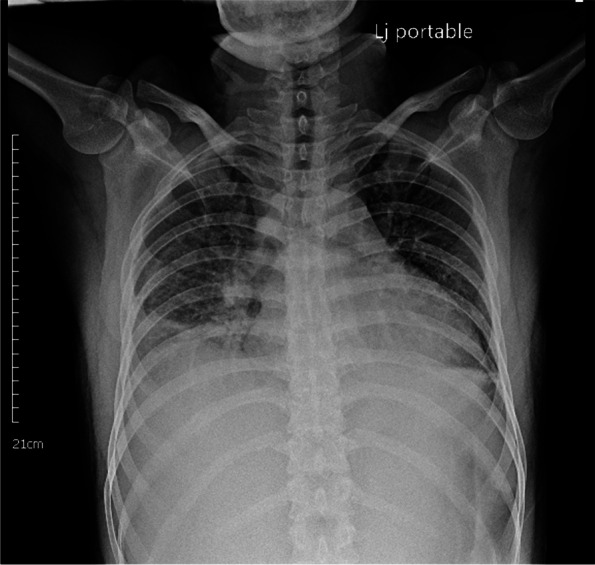
A chest radiography revealed bilateral blunt costodiaphragmatic angle and cardiomegaly

During the second and third days of the patient's hospitalization, he continued to experience intermittent fever of approximately 39 °C for three days, despite being treated with intravenous cefotaxime as an empirical antibiotic. He also suffered from persistent tachycardia ranging from 100/min to 140/min and chest distress with pleuritic chest pain. Shock was observed on the second night of admission, and the patient's white blood cell count increased, while hemoglobin and platelet levels decreased. Additionally, his procalcitonin and C-reactive protein levels were elevated, along with elevated troponin I and creatine kinase-myoglobin. A chest radiography revealed bilateral blunt costodiaphragmatic angle and cardiomegaly (Fig. 2). Bilateral blunting of the costophrenic angles and increased radiopacity in the right lower and middle lobes were observed, accompanied by cardiomegaly. Bilateral pleural effusion, with a predominant presence on the right side, was later confirmed by bedside echocardiography.), while an electrocardiogram showed sinus tachycardia without ST-segment and T-wave changes. A bedside echo confirmed an inferior vena cava diameter of 1.67 cm and bilateral pleural effusion. Due to his deteriorating condition, the patient's antimicrobial treatment was upgraded to intravenous teicoplanin with cefotaxime, and he was moved to the intensive care unit (ICU) because of his shock status. He was intubated and given mechanical ventilation support due to respiratory failure. In addition, he required a norepinephrine pump due to hemodynamic instability. Later, a cardiologist diagnosed the patient with myopericarditis-related cardiogenic shock based on the cardiac echo findings, which showed left ventricular global hypokinesis with 40% left ventricular ejection fraction and a small amount of pericardial effusion. On the third day, a stool polymerase chain reaction (PCR) panel revealed a positive result for *Campylobacter spp*. Oral Azithromycin followed by parenteral clarithromycin was administered to the patient. All other infectious workup tests, including blood culture, urine culture, FilmArray test for a pneumonia PCR panel, urine PCR and blood IgM for leptospirosis, and blood PCR, IgG, and IgM for Hanta virus and severe fever with thrombocytopenia syndrome, yielded negative results.

During the patient's stay in the ICU, they showed signs of kidney dysfunction, including reduced urine output, weight gain, and swelling in both legs that left a noticeable indentation when pressed. His hemoglobin and platelet levels dropped to their lowest points of 8.3 g/dL and 57 × 103/μl, respectively. Additionally, the patient's ferritin level was found to be 1008 ng/mL, and his triglyceride level had risen to 631 mg/dL.

**Fig. 3:**
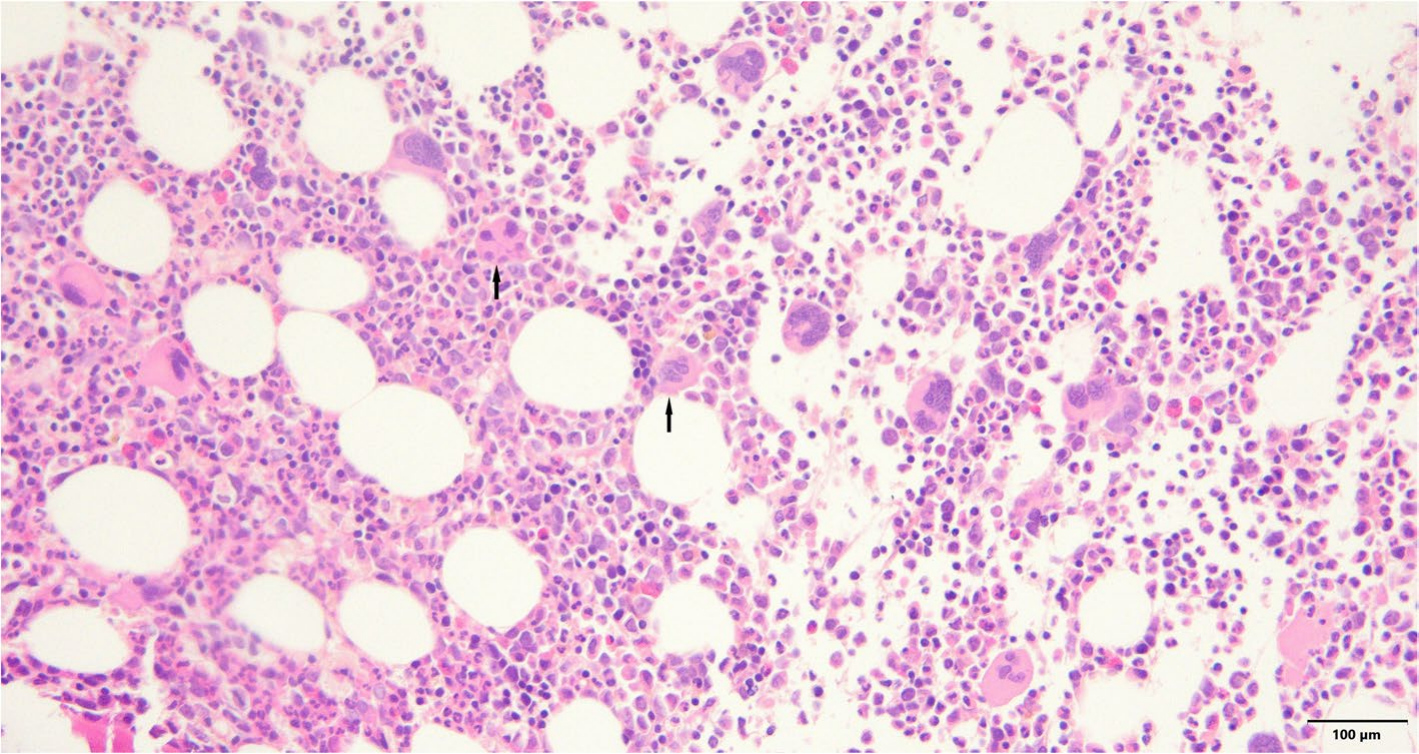
Pathology slide for the boen marrow biopsy

**Fig. 4:**
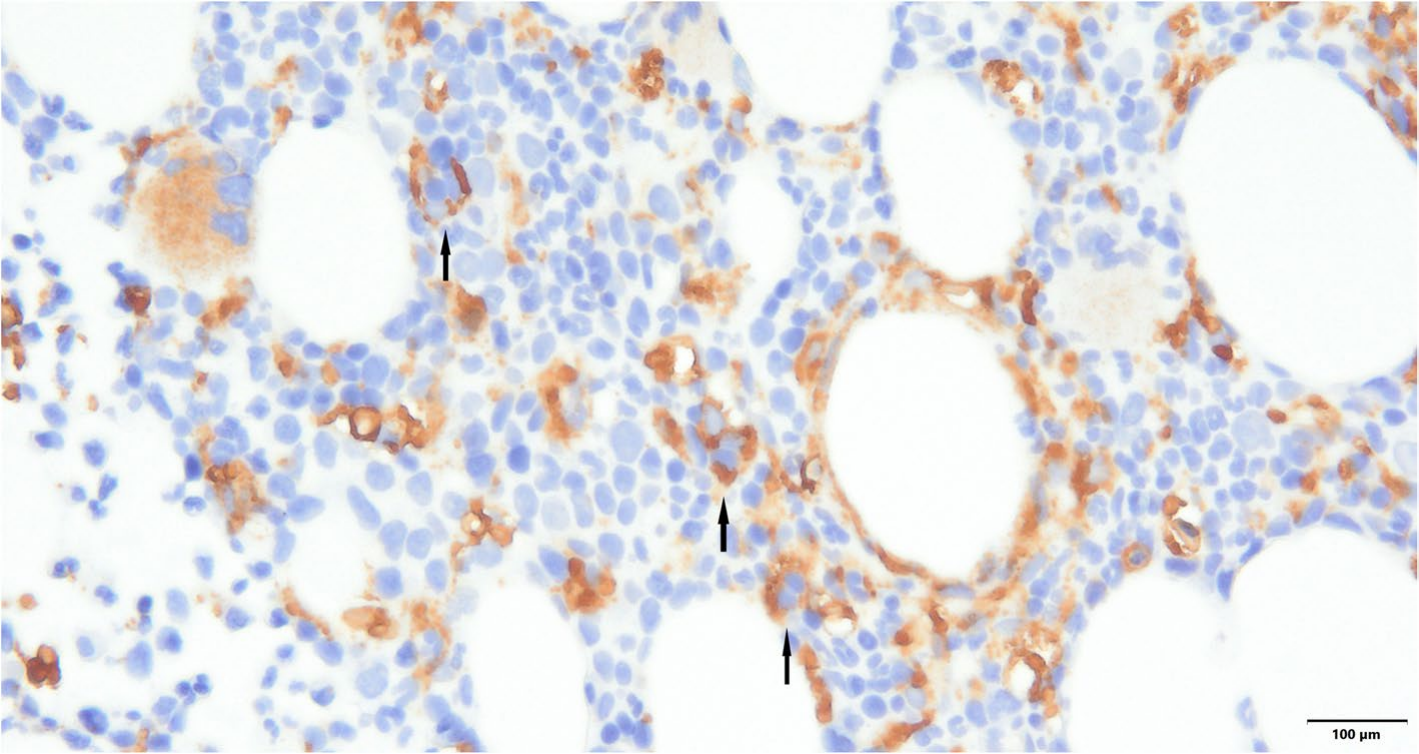
CD68 immunohistochemistry distinctly

On day 6, a bone marrow biopsy confirmed that the patient was suffering from hemophagocytosis (Fig. 3). The bone marrow aspirate was examined with hematoxylin and eosin stain, revealing significant phagocytosis of hematopoietic elements (arrows) and an increased number of activated macrophages at 400 × magnification.

(Fig. 4). CD68 immunohistochemistry distinctly highlights hemophagocytic histiocytes (arrows) in bone marrow biopsy specimens at 400 × magnification.), which led to a diagnosis of HLH. As a result, the patient was started on a course of dexamethasone 10 mg every 12 h.

Further tests were conducted to rule out other potential causes, such as HIV, Epstein‒Barr virus, and non-Hodgkin lymphoma, through blood and bone marrow examinations. The patient's persistent oliguria and high creatinine levels suggested sepsis and cardiorenal-related acute kidney injury (AKI). As a result, the patient underwent temporary hemodialysis on days 6, 7, and 9.

As the patient's condition improved under treatment, their dexamethasone dosage was gradually reduced, and they were weaned off the endotracheal tube. Their hemodialysis was also discontinued once their urine output increased and their creatinine levels decreased.

## Discussion

*Campylobacter* is a type of gram-negative bacteria that can lead to food poisoning and gastroenteritis in humans. Among the different species of *Campylobacter*, *Campylobacter jejuni* is the most prevalent, causing approximately 90% of human infections worldwide, followed by *Campylobacter coli* [6]. These bacteria are often transmitted through the consumption of undercooked poultry, pork, beef, unpasteurized milk, and contaminated water [7]. In most cases, the infection resolves on its own without any significant consequences. However, postinfectious complications such as reactive arthritis, irritable bowel syndrome, and Guillain Barré syndrome are sometimes observed [8]. Recently, there have been some reports suggesting that *Campylobacter* may also be linked to rare diseases such as HLH and myocarditis.

HLH is a severe condition that occurs when cytotoxic T cells, natural killer cells, and macrophages become overactive, resulting in hypercytokinemia and damage to multiple organs. This disorder can affect people of all ages and is divided into two categories: primary HLH, which results from genetic mutations that impair the function of cytotoxic T cells and natural killer cells, and secondary HLH, which results from underlying malignancy, infection, or autoimmune disorders. HLH can present with a range of clinical and laboratory features, including fever, splenomegaly, neurological dysfunction, coagulopathy, liver dysfunction, cytopenias, hypertriglyceridemia, hyperferritinemia, hemophagocytosis, and reduced natural killer cell activity [9]. Only two cases of HLH caused by *C. jejuni* have been reported thus far [10, 11].

To our knowledge, there have been no reported cases of *Campylobacter* causing both HLH and perimyocarditis. After ruling out other possible diagnoses, we concluded that *Campylobacter* infection triggered an autoimmune response, resulting in the development of both HLH and perimyocarditis in our patient. This case highlights the importance of considering HLH in patients with unexplained fever and pancytopenia, as well as the potential complications of *Campylobacter* infection. Although HLH and myocarditis caused by *Campylobacter* may be rare, our experience with treatment suggests that the prompt identification of HLH and myocarditis caused by *Campylobacter*, as well as the timely use of steroids and antibiotics for the treatment of HLH caused by *Campylobacter*, can lead to a good prognosis.

## Conclusion

This case report is the first documented instance of both HLH and perimyocarditis occurring simultaneously due to a *Campylobacter* infection. Our findings add to the existing knowledge regarding the link between *Campylobacter* infections and the effectiveness of steroid treatment for HLH caused by *Campylobacter*. As *Campylobacter*-related HLH and pericarditis are rare occurrences, further clinical studies and trials are necessary to improve our understanding of the underlying mechanisms and the role of *Campylobacter* in inducing pericarditis with HLH.

### Supplementary Information

**Supplementary material 1.**

## Data Availability

The datasets generated during and/or analyzed during the current study are not publicly available due to all data from this article being available exclusively at Tong Comprehensive Hospital, but are available from the corresponding author on reasonable request.

## Abbreviations

HLH: Hemophagocytic lymphohistiocytosis
ICU: Intensive care unit
AKI: Acute kidney injury
PCR: Polymerase chain reaction
