# Supplementary material for: Hemophagocytic lymphohistiocytosis and myopericarditis induced by campylobacter: a case report
Source: BMC Infect Dis. 2024 Apr 8;24:382. doi: 10.1186/s12879-024-09128-z (PMC11003181; doi:10.1186/s12879-024-09128-z)
Supplement: Supplementary file 1 — Supplementary material 1. [file 12879_2024_9128_MOESM1_ESM.docx]

**Additional file 1**

| Table 1 Laboratory Test Results | | | | | | | | | | | |
| --- | --- | --- | --- | --- | --- | --- | --- | --- | --- | --- | --- |
|  | WBC | RBC | Hb | Ht | MCV | PLT | Seg | Lym | Mono | Eosi | Baso |
| Normal Range | 4~10 | 4.5~5.5 | 13~17 | 41~51 | 80~100 | 140~520 | 35~75 | 20~55 | 1~12 | 1~5 | 0~1.6 |
| Unit | 10^3^/uL | 10^6^/uL | g/dL | % | fL | 10^3^/uL | % | % | % | % | % |
| Day 1 | 7.1 | 5.13 | 13.3 | 40.7 | 79.3 | 172 | 93.5 | 2 | 2.4 | 1.8 | 0.3 |
| Day 3 | 13.8 | 4.54 | 11.6 | 34.5 | 76 | 104 | 94 | 2.2 | 1.7 | 1.7 | 0.4 |
| Day 5 | 24 | 4.6 | 11.9 | 33.8 | 73.5 | 84 | 95.1 | 2.2 | 0.8 | 1.6 | 0.3 |
| Day 6 | 31.1 | 4.03 | 10.4 | 28.8 | 71.5 | 57 | 96.1 | 1.7 | 1 | 0.8 | 0.4 |
| Day 8* | 32.1 | 3.98 | 10.1 | 28.4 | 71.4 | 57 | 94.3 | 2.3 | 2.9 | 0.4 | 0.1 |
| Day 9 |  |  | 10.5 |  |  | 64 |  |  |  |  |  |
| Day 11 | 31.7 | 3.7 | 9.4 | 27.5 | 74.3 | 114 | 93.2 | 3.1 | 3.5 | 0.1 | 0.1 |
| Day 12 |  |  | 9.6 |  |  | 140 |  |  |  |  |  |
| Day 15 | 17.4 | 3.19 | 8.3 | 25.9 | 81.2 | 101 | 86.1 | 7.6 | 5 | 1 | 0.3 |
| Day 16 | 14.7 | 3.48 | 9 | 28.5 | 81.9 | 111 |  |  |  |  |  |
| Day 18 | 12.2 | 3.49 | 9 | 29.8 | 85.4 | 138 |  |  |  |  |  |

*On days 7 and 8, Dexamethasone was administered at a dosage of 10 mg every 12 hours. On days 9 and 10, the dosage was adjusted to 10 mg once daily in the morning plus 5 mg at bedtime. From days 10 to 12, Dexamethasone was administered at a dosage of 5 mg every 12 hours. On days 13 and 14, the dosage was further adjusted to 5 mg once daily. Starting from day 14, the patient was kept on Dorison at a dosage of 4 mg until the outpatient department visit, with a gradual tapering plan in place.

WBC, White Blood Cell Count; RBC, Red Blood Cell Count; Hb, Hemoglobin;
Ht, Hematocrit; MCV, Mean Corpuscular Volume; PLT, Platelet Count;
Seg, Segmented Neutrophils; Lym, Lymphocytes; Mono, Monocytes; Eosi, Eosinophils; Baso, Basophils.

| **Table 2** Laboratory Test Results | | | | | | | | | | | |
| --- | --- | --- | --- | --- | --- | --- | --- | --- | --- | --- | --- |
|  | Glucose | r-GT | SGOT | SGPT | ALK-P | D-bil | T-bil | ALB | TG | CHO | LDH |
| Normal Range | 70~100 | 16~73 | 8~40 | 8~40 | 104~338 | 0~0.5 | 0.2~1.2 | 3.4~5.3 | 0~150 | 0~220 | 106~221 |
| Unit | mg/dL | IU/L | IU/L | IU/L | IU/L | mg/dL | mg/dL | g/dL | mg/dL | mg/dL | U/L |
| Day 1 | 174 | 179 | 66 | 104 |  | 1.2 |  |  |  |  |  |
| Day 2 |  |  |  |  |  |  | 2.4 |  |  |  | 203 |
| Day 3 |  | 126 | 26 | 55 | 337 |  | 3.6 | 2.7 |  |  | 227 |
| Day 5 |  | 75 | 48 | 45 |  | 3.6 | 5.4 |  | 631 | 103 |  |
| Day 7 |  |  | 28 | 33 |  | 3.8 | 5.4 | 2.4 |  |  |  |
| Day 12 |  |  | 14 | 30 |  | 0.5 | 1.2 | 2.5 |  |  |  |
| Day 16 |  |  |  |  |  |  | 1 |  |  |  |  |
| Day 19 |  |  | 23 |  |  |  |  |  |  |  | 157 |

r-GT, Gamma-Glutamyl Transferase; SGOT, Serum Glutamic Oxaloacetic Transaminase; SGPT, Serum Glutamic Pyruvic Transaminase; ALK-P, Alkaline Phosphatase; D-bil, Direct Bilirubin; T-bil, Total Bilirubin; ALB, Albumin;
TG, Triglycerides; CHO, Cholesterol; LDH, Lactate Dehydrogenase.

| **Table 3** Laboratory Test Results | | | | | | | | |
| --- | --- | --- | --- | --- | --- | --- | --- | --- |
|  | BUN | Crea | eGFR | UA | Na | K | Ca | P |
| Normal Range | 7~22 | 0.5~1.3 | 100~140 | 3.6~8.0 | 137~150 | 3.5~5.3 | 8.4~10.2 | 2.7~4.5 |
| Unit | mg/dL | mg/dL |  | mg/dL | meq/L | meq/L | mg/dL | mg/dL |
| Day 1 | 18 | 1.15 | 80.5 |  | 131 | 3.6 |  |  |
| Day 3 | 16 | 1.39 | 64.7 | 4.8 | 129 | 3.6 |  |  |
| Day 4 |  |  |  | 5.1 |  |  | 6.7 | 2.2 |
| Day 5 | 40 | 3.28 | 24 |  |  | 2.9 | 7.3 | 3.4 |
| Day 7 | 69 | 6.03 | 11.9 |  | 134 | 3.5 | 7 | 2.9 |
| Day 12 | 89 | 2.49 | 33 |  | 147 | 4.6 | 7 |  |
| Day 13 |  |  |  |  | 147 | 5.1 | 7.6 |  |
| Day 14 | 71 | 1.54 | 57.5 |  | 140 | 5.3 | 7.4 |  |
| Day 15 |  |  |  |  |  | 5.7 |  |  |
| Day 16 | 48 | 1.24 | 73.8 |  | 135 | 4.9 |  |  |
| Day 17 |  |  |  |  |  | 4.4 |  |  |
| Day 19 | 27 | 0.83 | 117.3 |  | 135 | 4.3 |  |  |

BUN, Blood Urea Nitrogen; Crea, Creatinine; eGFR, Estimated Glomerular Filtration Rate; UA, Uric Acid; Na, Sodium; K, Potassium; Ca, Calcium; P, Phosphorus.
